# Assessment of Exercise-Stimulated Irisin in Kidney Disease: A Systematic Review

**DOI:** 10.2174/0118715303381483250629164853

**Published:** 2025-09-19

**Authors:** Enzo Shintaku, Davi Vantini, João Gabriel Bicudo Ting, Rubén David dos Reis Zuniga, Glaucia Luciano da Veiga, Beatriz da Costa Aguiar Alves, Jéssica Freitas Encinas, Fernando Luiz Affonso Fonseca

**Affiliations:** 1 Clinical Analysis Laboratory, Centro Universitário FMABC, Santo André - Brazil;; 2 Department of Pharmaceutical Sciences, Universidade Federal de São Paulo, Diadema - Brazil

**Keywords:** Irisin, physical exercise, renal disease, systematic review, metabolic disease, type 2 diabetes, antiapoptotic effects

## Abstract

**Introduction:**

Irisin is a hormone synthesized by skeletal muscle cells in response to physical exercise. It has been linked to various health benefits, including improved insulin sensitivity, fat burning, reduced inflammation, and potential protection against metabolic diseases, such as obesity and type 2 diabetes. This review explores the role of irisin, stimulated by physical exercise, in kidney diseases.

**Methods:**

A comprehensive review was conducted using SciELO, PubMed, Scopus, Web of Science, and EMBASE. Five articles were selected based on pre-established eligibility criteria. These studies used animal experiments, assessing irisin predominantly through Western blotting or ELISA, with aerobic exercise protocols, mainly treadmill running.

**Results:**

The results consistently showed an increase in irisin levels in response to physical exercise in animal models with kidney diseases.

**Discussion:**

Aerobic exercise increased plasma irisin expression, with better outcomes observed with low to medium-intensity training. Irisin demonstrated therapeutic potential by reducing renal cysts, inhibiting epithelial-mesenchymal transition, decreasing markers of diabetic nephropathy, and restoring autophagy in podocytes. Additionally, it activated the AMPK-Sirt1-PGC-1α pathway, suggesting antioxidant and antiapoptotic effects.

**Conclusion:**

The studies reviewed suggested that aerobic exercise increased irisin levels in animal models of kidney diseases, showing potential therapeutic effects for renal health.

## 1 INTRODUCTION

Irisin is a hormone discovered in 2012 that has attracted great scientific interest due to its potential role in several physiological functions [1, 2]. It is composed of 112 amino acids and its production is stimulated by the peroxisome proliferator-activated receptor gamma coactivator 1-alpha (PGC-1α), which is induced by physical exercise. When PGC-1α is activated, it promotes the expression of the FNDC5 protein (protein 5 containing the fibronectin type III domain), which is further cleaved to release irisin into the bloodstream [1].

Initially, irisin was identified as a myokine produced by skeletal muscle tissue in response to physical exercise, being released into the bloodstream to act on other organs. Recent studies, however, suggest that other cell types, such as neurons and adipocytes, can also synthesize it [3]. In these cells, irisin contributes to the rescue of memory in patients with Alzheimer's [4], regulates energy metabolism, promotes the browning of adipose tissues, stimulates the expression of UCP1 (Uncoupling Protein 1), increases energy expenditure, and improves obesity and glucose metabolism [3]. In addition, irisin has been linked to several health benefits, including protection against cardiovascular disease and liver injury, anti-inflammatory action, and improved insulin sensitivity [1, 3-6].

Current research indicates that one of irisin's roles is to act as a therapeutic marker in kidney diseases (KDs) [7]. Specifically, among them, acute kidney disease (AKD), characterized by a sudden loss of kidney function, is considered one of the most impactful, as it is a major risk factor for the development and progression of chronic kidney disease (CKD), which is currently one of the most prevalent kidney disorders. Clinically, the main causes of acute kidney disease include renal ischemia-reperfusion, nephrotoxic drugs, and sepsis. Pathologically, it is characterized by injury to renal proximal tubular epithelial cells, with mitochondrial damage, disturbed energy metabolism, epigenetic regulation, and inflammation being the main mechanisms implicated in its pathogenesis [3].

As this disease progresses, it leads to an almost irreversible condition known as chronic kidney disease, in which the kidneys gradually lose their ability to filter waste and fluids from the blood [8]. Several conditions contribute to the development of this pathology, including diabetes mellitus, high blood pressure, obesity and cardiovascular diseases. Considering this, the aspects that can be favorable in the prevention and treatment of this disease have been increasingly analyzed [9-11]. One of the possible factors observed is the decreased level of irisin in patients with end-stage kidney disease, suggesting that the decrease in serum concentration of this hormone, concomitantly with the progression of the disease, may predict renal function [3].

Thus, the increase in exercise-induced irisin expression may benefit various organs of the human body, especially in the context of kidney pathologies. Thus, based on the above, this study aims to analyze the involvement of this hormone, stimulated by physical exercise, in kidney diseases.

## 2 METHODS

Through a systematic review and analysis of scientific articles, we conducted a study that aligns with recent research on the role of exercise-stimulated irisin in kidney disease. Using the research platforms, such as SciELO, PubMed, Scopus, Web of Science and EMBASE, a compendium of articles was created that examined the association of the aforementioned factors. All were reviewed and categorized according to the PICO and PRISMA methodologies, with the purpose of reporting the existing studies on the subject [12-14].

**Fig. (1):**
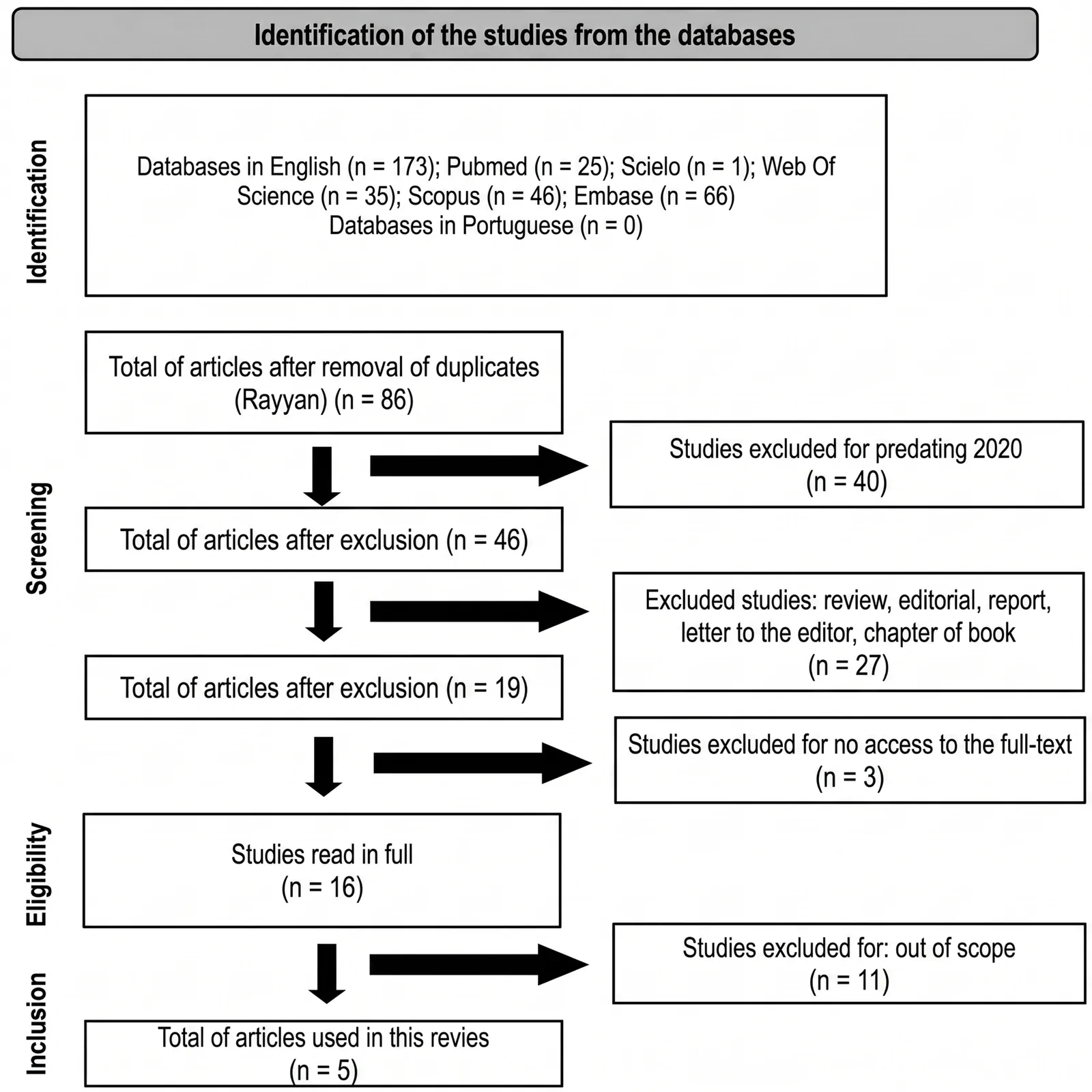
A PRISMA Flowchart: Selection of studies for systematic reviews [12].

First, the PICO methodology was employed. The research investigated the population composed of individuals diagnosed with kidney disease, with the aim of evaluating the influence of different interventions on the progression of the condition. The intervention of interest is the effect of exercise-stimulated irisin and its potential impact on kidney disease. Individuals who led a sedentary life were considered a comparison group. The primary endpoint to be analyzed was the efficacy of irisin, both as a biological marker and as a possible treatment for kidney disease, investigating whether the presence and action of this hormone can improve the clinical outcomes of patients. The central hypothesis is that physical exercise, by stimulating the production of irisin, contributes to the modulation of kidney disease, compared to the negative impact of a sedentary lifestyle. Next, the study selection process was performed using the PRISMA flowchart (Fig. **1**).

The keywords “irisin”, “physical exercise” and “renal disease” used in the databases were the most commonly used terms in scientific articles. Thus, variations and combinations of these health descriptors (https://decs.bvsalud.org/) in English were used in database searches; for the EMBASE platform, ('irisin'/exp OR irisin OR fndc5) AND ('exercise'/exp OR exercise OR 'aerobic exercise'/exp OR 'aerobic exercise' OR (aerobic AND ('exercise'/exp OR exercise)) OR 'resistance exercise training'/exp OR 'resistance exercise training' OR (('resistance'/exp OR resistance) AND ('exercise'/exp OR exercise) AND ('training'/exp OR training)) OR 'physical exercise'/exp OR 'physical exercise' OR (physical AND ('exercise'/exp OR exercise))) AND ('chronic kidney disease'/exp OR 'chronic kidney disease' OR (chronic AND ('kidney'/exp OR kidney) AND ('disease'/exp OR disease)) OR 'renal function'/exp OR 'renal function' OR (('renal'/exp OR renal) AND ('function'/exp OR function)) OR 'renal failure'/exp OR 'renal failure' OR (('renal'/exp OR renal) AND ('failure'/exp OR failure)) OR 'kidney failure'/exp OR 'kidney failure' OR (('kidney'/exp OR kidney) AND ('failure'/exp OR failure)) OR 'renal dysfunction'/exp OR 'renal dysfunction' OR (('renal'/exp OR renal) AND dysfunction)) were used and for the PubMed, Web of Science, SciELO, and Scopus platform, ((irisin) OR (fndc5)) AND ((exercise) OR (aerobic AND exercise) OR (resistance AND exercise AND training) OR (physical AND exercise)) AND ((chronic AND kidney AND disease) OR (renal AND function) OR (renal AND failure) OR (kidney AND failure OR (renal AND dysfunction)) were used. In addition, various terms and combinations in Portuguese were also used on the PubMed, SciELO, Web of Science, Scopus, and EMBASE platforms, such as ((irisina) OR (FNDC5)) AND ((exercício) OR (exercício físico) OR (exercício aeróbico) OR (treinamento) OR (treinamento de exercício de resistência)) AND ((doença renal crônica OR (função renal) OR (falha renal) OR (falha do rim) OR (disfunção renal)).

A total of 173 studies on the subject were found in the databases of PubMed (n = 25), SciELO (n = 1), Web of Science (n = 35), Scopus (n = 46), and EMBASE (n = 66). In the identification stage, all studies were centralized in the Rayyan [13] (https://rayyan.ai/reviews/968917). Of these, 87 studies were duplicated among these databases and were excluded. In the next stage of screening and eligibility of the remaining 86 articles, 40 studies by year and 27 studies were excluded by the automatic “article” filter of the Web of Science, Scopus and EMBASE databases, in addition to the manual filter in PubMed, which excluded articles from reviews. In addition, 3 studies were excluded because they did not acquire full-text access. Finally, after reading the remaining 16 articles in full-text, 11 studies were excluded because they did not detail the 3 keywords of the theme (out of scope), and only 5 studies were included.

From the 5 selected articles, a table was made including the following information: title, authors and year of publication, journals, sample, study design, method, results and conclusions.

All the articles included in the systematic review used animal experiments, and some of them showed beneficial effects of physical exercise associated with irisin, which was predominantly analyzed using the Western blotting method and ELISA. In addition, most studies employed an aerobic exercise protocol, which consisted primarily of a treadmill running model.

## 3 RESULTS

The results obtained consistently demonstrated an increase in irisin levels in response to physical exercise in animal models with adverse renal conditions, as reported in the summary of the articles used in the systematic review (Table **1**).

## 4 DISCUSSION

The studies analyzed investigated the effects of physical exercise and irisin in different animal models and kidney cells, with a focus on kidney diseases. In SHR rats, low- and medium-intensity aerobic exercise significantly increased the expression of FNDC5 (fibronectin type III domain-containing protein 5) and irisin in the kidneys, improving renal function and inhibiting Ang II-induced EMT (Epithelial-Mesenchymal Transition) in HK-2 cells. High-intensity exercise did not have a similar effect, possibly due to the activation of the STAT3 pathway by irisin [7]. In diabetic rats, eight weeks of aerobic exercise reduced markers of diabetic nephropathy and increased AMPK activation and FNDC5-irisin expression, with recombinant irisin decreasing the expression of collagen IV and fibronectin in HK-2 cells exposed to high glucose. These effects suggest a crucial role of irisin and AMPK in exercise-induced renal protection [4].

It was also found that mice with myocardial infarction submitted to aerobic exercise showed reduced expression of ALCAT1 (acyl-CoA acyltransferase 1), oxidative stress and apoptosis in the kidney. In this study, six-week aerobic training and ALCAT1 knockout reduced oxidative stress and apoptosis in infarction-induced kidney injury. Treatment with irisin activated the AMPK-Sirt1-PGC-1α pathway, alleviating oxidative stress and apoptosis. This suggests aerobic exercise inhibits oxidative stress-induced apoptosis in impaired kidneys, in part by activating the FNDC5/Irisin-AMPK-Sirt1-PGC-1α pathway and inhibiting ALCAT1 expression [9]. Physical exercise also slowed the progression of renal dysfunction and cyst growth in PCK rats, in addition to increasing plasma excretion of irisin. These results indicate the therapeutic potential of chronic exercise on renal dysfunction and cyst growth, although further studies may be necessary to clarify the effects of irisin [10].

In diabetic mice, overexpression of PGC-1α (peroxisome proliferator-activated receptor gamma coactivator 1-alpha) in skeletal muscle, a model that mimics the effects of muscle exercise, increased irisin levels, promoted autophagy in podocytes, and inhibited the PI3K/AKT/mTOR pathway. Treatment with recombinant irisin restored autophagy and reduced kidney injury, evidencing the renoprotective mechanism of exercise in diabetic kidney disease. These findings suggest that irisin plays essential roles in mediating the renal benefits of physical exercise, with potential implications for the treatment of kidney disease and diabetes [11].

In summary, it has been observed that aerobic physical exercise has the effect of increasing plasma irisin expression, with more advantageous results observed in low- and medium-intensity training [7]. This hormone, in turn, demonstrates therapeutic potential in several conditions, including the reduction of renal cysts, the inhibition of the epithelial-mesenchymal transition, the reduction of diabetic nephropathy markers, and the restoration of autophagy in podocytes. In addition, irisin was found to be able to activate cell signaling pathways, such as the AMPK-Sirt1-PGC-1α pathway, thus exerting antioxidant and antiapoptotic effects [4, 7, 9, 10].

## CONCLUSION

Aerobic exercise has been shown to increase irisin levels in animal models with kidney disease. However, there is a notable gap in research regarding the effects of resistance training on irisin levels and kidney health. Further studies are essential to elucidate the renoprotective mechanisms of irisin and to assess its efficacy and safety in human populations.

## AUTHORS’ CONTRIBUTIONS

The authors confirm their contribution to the paper as follows: study conception and design: ES and FLAF; methodology: JT and RRZ; visualization: BCAA and JE; validation: DV; writing, reviewing, and editing: GV. All authors reviewed the results and approved the final version of the manuscript.

## Figures and Tables

**Table 1:** Summary of the articles included in the systematic review.

**Articles**	**Authors and Year **	**Journals**	**Type of Study**	**Sample**	**Method**	**Results**	**Conclusion **
Aerobic exercise inhibits renal EMT by promoting irisin expression in SHR	Minghao Luo, *et al* - 2023	iScience	Preclinical study	Thirty-two male spontaneously hypertensive rats (SHR), eight weeks old and healthy, including 8 sedentary SHR and 8 sedentary Wistar-Kyoto rats of the same age. Additionally, human renal proximal tubule epithelial cells (HK-2) were cultured and supplemented.	Twenty-four SHR were subjected to an adaptation period consisting of five days of running sessions at 60 minutes per day, at a speed of 8 m/min, between 3:00 PM and 5:00 PM. Subsequently, they were divided into three groups and exposed to treadmill exercise at 0° inclination with varying intensities: low-intensity aerobic training (30–40% of maximum capacity, 14 m/min), moderate-intensity (45–55%, 20 m/min), and high-intensity (60–70%, 26 m/min) for 60 minutes per day, between 3:00 PM and 5:00 PM, over 14 weeks. HK-2 cells were incubated with different concentrations of irisin in the absence or presence of angiotensin II for 24 hours. Irisin levels were analyzed using an ELISA kit.	The expression of FNDC5 and irisin in the kidneys of the low-, moderate-, and high-intensity training groups was significantly higher than in the kidneys of the sedentary SHR group. Low- and moderate-intensity training significantly improved renal function and inhibited epithelial-to-mesenchymal transition (EMT) in SHR, a process that may lead to renal fibrosis, while high-intensity training had no effect in this regard. Irisin demonstrated a therapeutic effect on the morphological alterations induced by Ang II in HK-2 cells.	Aerobic exercise contributed to the synthesis and release of irisin. This hormone exerted a therapeutic effect by inhibiting Angiotensin II-induced EMT in HK-2 renal cells *in vitro*. Activation of the STAT3 pathway by irisin may explain the differential effects observed with high-intensity exercise.
Renal protection induced by physical exercise may be mediated by the irisin/AMPK axis in diabetic nephropathy	Guilherme Pedron Formigari, *et al* - 2022	Nature	Preclinical study	One hundred male Wistar Hannover (HanUnib) rats, eight weeks old, were divided into two experiments: one experiment included 13 non-diabetic, 13 induced-diabetic sedentary, and 9 induced-diabetic exercised rats. The second experiment included 8 induced-diabetic sedentary rats,	Rats were adapted to exercise on a motorized treadmill with a 0° incline for five days. Aerobic training began after diabetes induction, lasting four weeks, five days a week, at 60% exhaustion speed. Exercise duration gradually increased (15, 30, 45, 60 minutes). Training intensity was adjusted	Eight weeks of aerobic exercise in diabetic rats reduced diabetic nephropathy markers and were associated with renal and muscular activation of AMPK (AMP-activated protein kinase) and increased FNDC5-irisin expression. Treatment with CycloRGDyK abolished the beneficial effects of aerobic exercise in diabetic rats.	Aerobic exercise provides renoprotection by reducing markers of inflammation and glomerular fibrosis, with an increase in muscle and serum irisin levels and renal AMPK activity. Treatment with CycloRGDyK corroborated the protective effect of irisin. The use of recombinant irisin in *in vitro* experiments and serum from exercised diabetics demonstrated protection of HK-2 cells from the adverse effects of elevated glucose, indicating that the irisin/AMPK axis may mediate exercise-induced renal protection in diabetes.
				7 induced-diabetic sedentary rats treated with CycloRGDyK (an irisin blocker), 8 induced-diabetic exercised rats, and 7 induced-diabetic exercised rats treated with CycloRGDyK. Additionally, human HK-2 renal proximal tubular epithelial cells were cultured and supplemented.	based on incremental load tests performed at baseline, after four, and at eight weeks. HK-2 cells were exposed to normal and high glucose (HG), treated with three concentrations of recombinant irisin. Additional HK-2 cells were cultured in HG with 4% human serum from non-diabetic, sedentary, and exercised diabetic patients. Serum irisin was measured *via* ELISA.	Treatment of HK-2 cells with high glucose (HG) and recombinant irisin reduced the elevated expression of collagen IV and fibronectin and increased AMPK activity. Treatment of HK-2 cells exposed to HG and serum from exercised diabetic individuals showed a significant reduction in collagen IV and fibronectin expression.	
Aerobic exercise alleviates oxidative stress-induced apoptosis in kidneys of myocardial infarction mice by inhibiting ALCAT1 and activating FNDC5/Irisin signaling pathway	Fangnan Wu, *et al* - 2020	Free Radical Biology and Medicine	Preclinical study	Wild-type male C57/BL6 mice (8 weeks old) were randomly divided into a sham operation group (S), myocardial infarction group (MI), and exercise after myocardial infarction group (ME). Male alcat1 knockout (KO) mice were randomly assigned to the KS (knockout with sham myocardial infarction), KMI (knockout with myocardial infarction), and KME (exercise in knockout with myocardial infarction) groups, with 10 mice in each group. Normal rat kidney (NRK) cells were cultured in a cell incubator.	The ME and KME mice underwent moderate-intensity aerobic exercise. In the first week, treadmill training was performed. Mice were trained at 10 m/min for 10 minutes on the first day, with an increase of 10 minutes per day until the fifth day. Subsequently, regular physical training for five weeks was conducted at 10 m/min for 60 minutes per day, five days a week, for six weeks. TUNEL staining was used to detect the levels of cellular apoptosis in kidney tissues, assay kits were used to analyze oxidative stress, and Western blotting technique was applied to analyze irisina. NRK cells were treated with H2O2, rhIrisin, AICAR, EX527, and transfected with lentivirus containing the alcat1 gene.	Aerobic exercise reduced the high expression of ALCAT1 in the kidneys of mice with MI. ALCAT1 (Acyl-CoA Lysocardiolipin Acyltransferase 1) was positively correlated with oxidative stress and apoptosis. Increased expression of Irisin was associated with improved renal function in the kidneys of MI mice after aerobic exercise. Renal fibrosis area and renal injury scores were increased in the MI group compared to the S group but were markedly reduced in the ME group, resembling the results in alcat1 KO mice. AICAR, the AMPK agonist, accelerated the inhibitory effect of rhIrisin on oxidative stress-induced apoptosis in NRK cells. AMPK-activated Sirt1 regulated energy metabolism.	Aerobic exercise inhibited oxidative stress-induced apoptosis in kidneys damaged by myocardial infarction, partly through the activation of the FNDC5/Irisin-AMPK-Sirt1-PGC-1α signaling pathway and the inhibition of ALCAT1 expression, although the underlying mechanisms are not yet fully understood.
Chronic exercise protects against the progression of renal cyst growth and dysfunction in rats with polycystic kidney disease	Jiahe Qiu, *et al* - 2021	Medicine & Science in Sports & Exercise	Preclinical study	Thirty 6-week-old male rats, consisting of ten sedentary polycystic kidney (PCK) rats, ten PCK rats subjected to physical exercise, and ten Sprague-Dawley (SD) control rats.	Twenty PCK rats and ten SD rats were used, with ten PCK rats subjected to moderate treadmill exercise for 12 weeks, starting with 10 minutes/day at 20 m/min and gradually increasing to 60 minutes/day at 28 m/min. After euthanasia, the rats were sacrificed, their kidneys were excised, decapsulated, and analyzed. Plasma irisin levels were measured using an enzyme-linked immunosorbent assay (ELISA) kit.	Chronic exercise in rats inhibited the increased urinary excretion of L-FABP (Liver-type Fatty Acid Binding Protein). This training also suppressed excessive cell proliferation, with negative regulation of the cAMP/B-Raf/ERK and mTOR/S6 pathways in renal epithelial cells. Additionally, chronic physical exercise reduced proteinuria and plasma creatinine levels, and attenuated glomerulosclerosis and podocyte injury. Chronic exercise also increased plasma irisin levels.	Chronic physical exercise may delay cyst growth, glomerular damage, and renal fibrosis, possibly by negatively regulating the cAMP/B-Raf/ERK and mTOR/S6 pathways and reducing Ki-67 positive cells. It may also alleviate proximal tubular stress and tubulointerstitial disturbances in PCK rats by decreasing urinary excretion of L-FABP.
Irisin ameliorates diabetic kidney disease by restoring autophagy in podocytes	Weiyan Lai, *et al* - 2023	FASEB Journal: official publication of the Federation of American Societies for Experimental Biology	Preclinical study	Male diabetic mice (db/db) and control littermates (db/m) at twelve weeks of age. Diabetic mice overexpressing mPGC-1α (db/db mPGC-1α+) and non-diabetic control mice (mPGC-1α+ db/m) as well as transgenic C57BL/6 mice with muscle-specific mPGC-1α, serving as an effective model to mimic the effects of muscle exercise. Plasma samples from 26 healthy volunteers and 76 patients with type 2 diabetes, with (n = 47) or without renal damage (n = 29). Renal tissues from type 2 diabetic patients, control renal tissues, and podocytes were used for analysis.	Four groups of 6 db/db and db/m mice received recombinant irisin or sterile phosphate-buffered saline (PBS) intraperitoneally for eight weeks. Irisin-treated groups received a weekly dose of 100 μg/kg. After treatment, at 24 weeks of age, the mice were anesthetized and sacrificed to collect blood and renal cortex tissue samples. Human podocytes were cultured with recombinant irisin (500 ng/mL) under normal glucose (5.5 mmol/L) and high glucose (30 mmol/L) conditions for 48 hours. Plasma irisin levels were measured using a recombinant ELISA kit.	The analysis revealed that irisin restored autophagy in podocytes by inhibiting the abnormal activation of the PI3K/AKT/mTOR signaling pathway. Irisin treatment significantly increased the number of autophagosomes in podocytes, reversing the number of these structures in cells exposed to high glucose to levels comparable to those observed in control cells. In mice, skeletal muscle PGC-1α overexpression increased irisin levels, resulting in a higher number of autophagosomes in the podocytes of db/db mPGC-1α+ mice. Irisin did not reduce blood glucose levels in mice.	Treatment with recombinant irisin in db/dbA mice and skeletal muscle overexpression of mPGC-1α highlighted the renoprotective mechanism of exercise through the regulation of podocyte autophagy mediated by irisin. The underlying mechanisms of the exercise benefits in diabetic kidney disease remain undefined.

## Data Availability

The data sets used and/or analysed during this study are available from the corresponding author upon request.

## LIST OF ABBREVIATIONS

UCP1: Uncoupling Protein 1
KDs: Kidney Diseases
AKD: Acute Kidney Disease
CKD: Chronic Kidney Disease
ALCAT1: Acyl-CoA Acyltransferase 1

## STANDARDS OF REPORTING

PRISMA guidelines and methodology were followed.
